# Coral probiotics induce tissue-specific and putative beneficial microbiome restructuring in a coral-dwelling fish

**DOI:** 10.1093/ismeco/ycaf052

**Published:** 2025-03-22

**Authors:** Joao Gabriel Duarte Rosado, Nathalia Delgadillo-Ordoñez, Matteo Monti, Viktor Nunes Peinemann, Chakkiath Paul Antony, Ahmed Alsaggaf, Inês Raimundo, Darren Coker, Neus Garcias-Bonet, Francisca García, Raquel Silva Peixoto, Susana Carvalho, Michael L Berumen

**Affiliations:** Biological and Environmental Science and Engineering Division (BESE), King Abdullah University of Science and Technology (KAUST), Thuwal, Makkah, 23955-6900, Saudi Arabia; Biological and Environmental Science and Engineering Division (BESE), King Abdullah University of Science and Technology (KAUST), Thuwal, Makkah, 23955-6900, Saudi Arabia; Biological and Environmental Science and Engineering Division (BESE), King Abdullah University of Science and Technology (KAUST), Thuwal, Makkah, 23955-6900, Saudi Arabia; Biological and Environmental Science and Engineering Division (BESE), King Abdullah University of Science and Technology (KAUST), Thuwal, Makkah, 23955-6900, Saudi Arabia; Biological and Environmental Science and Engineering Division (BESE), King Abdullah University of Science and Technology (KAUST), Thuwal, Makkah, 23955-6900, Saudi Arabia; Biological and Environmental Science and Engineering Division (BESE), King Abdullah University of Science and Technology (KAUST), Thuwal, Makkah, 23955-6900, Saudi Arabia; Biological and Environmental Science and Engineering Division (BESE), King Abdullah University of Science and Technology (KAUST), Thuwal, Makkah, 23955-6900, Saudi Arabia; Biological and Environmental Science and Engineering Division (BESE), King Abdullah University of Science and Technology (KAUST), Thuwal, Makkah, 23955-6900, Saudi Arabia; Biological and Environmental Science and Engineering Division (BESE), King Abdullah University of Science and Technology (KAUST), Thuwal, Makkah, 23955-6900, Saudi Arabia; Biological and Environmental Science and Engineering Division (BESE), King Abdullah University of Science and Technology (KAUST), Thuwal, Makkah, 23955-6900, Saudi Arabia; Biological and Environmental Science and Engineering Division (BESE), King Abdullah University of Science and Technology (KAUST), Thuwal, Makkah, 23955-6900, Saudi Arabia

**Keywords:** one health, coral probiotics, reef fish, risk assessment, microbiome

## Abstract

The ongoing fourth mass global coral bleaching event reinforces the need for active solutions to support corals through this major crisis. The use of beneficial microorganisms for corals (BMCs) offers a promising nature-based solution to rehabilitate coral’s dysbiotic microbiomes. While the benefits to corals are increasingly recognized, the impacts on associated reef organisms, such as fish, remain unexplored. This study investigated the effects of BMCs on the tissue-associated microbiomes of *Dascyllus abudafur* (*Pomacentridae*), a damselfish that lives closely associated with coral colonies. Over three months, we applied BMCs three times per week to healthy *Pocillopora verrucosa* colonies in the central Red Sea and analyzed the resultant changes in the inhabiting fish’s microbiomes. Our findings reveal significant, tissue-specific shifts in bacterial communities, particularly skin and gut, with moderate changes observed in gills. Notably, putative fish beneficial bacteria such as *Mitsuokella* spp. were enriched in the skin, while various Firmicutes taxa increased in the gut. There was also a marked decrease in potential fish pathogens. This study highlights the potential extended benefits of BMCs on coral reef fish and sets a foundation for understanding the broader ecological interactions between BMCs and reef-associated organisms.

## Introduction

Coral reefs are hotspots of marine biodiversity facing unprecedented threats in the current geological era, often named Anthropocene [1]. These key ecosystems increasingly undergo catastrophic bleaching events [2], where the coral host expels its photosynthetic algae symbionts, leading to coral starvation and, eventually, mortality [3,4]. These coral bleaching and mortality events have cascading effects on the ecological balance of the reef-associated fauna (i.e. fish), impacting the population dynamics, community structure, and behavior [5]. As the risk of climate inaction threatens the loss of most coral reef systems worldwide in the decades ahead [6], strategies aimed at reducing and mitigating CO_2_ emissions and addressing local impacts are crucial [7–9]. Nowadays, a plethora of active restoration strategies are being explored and rapidly developed, with the ultimate goal of restoring and retaining healthy coral reefs while the aimed reduction of global and local stressors is achieved [10]. One consists of applying beneficial microbial therapies to boost coral’s health and resilience against environmental change [11], one of the leading microbial solutions recently aligned as highly promising to mitigate climate change causes and consequences [12]. Like all metazoans, corals host an array of microorganisms comprising bacteria, fungi, archaea, viruses, microalgae, endoliths, and protists, forming the coral microbiome [13]. Healthy coral microbiomes are critical for their host’s ability to adapt to environmental changes more rapidly than natural selection and mutations would allow [14]. However, various synergistic sources of stress induce a dysbiotic state in corals, where the healthy microbiome status is replaced by a pathogenic assemblage [3].

Hence, the stewardship of microbiomes through the addition of beneficial microorganisms, also known as Beneficial Microorganisms for Corals (BMCs) [11], has been demonstrated to boost the health and resilience of their coral host by averting adverse effects from increasing temperatures and pathogens [15–17]. Recently, a pioneering study successfully reshaped the coral microbiome *in situ* by inoculating putative BMCs in healthy *Pocillopora verrucosa* coral colonies, enriching key beneficial bacteria while decreasing potential pathogens in the coral microbiome [18]. In this study, no detectable off-target effects were observed in seawater and sediment bacterial communities surrounding the coral, providing crucial evidence of the potential safe application of coral probiotics in reef ecosystems and their restructuring effect on coral-associated microbiomes. However, the enrichment of BMCs in other reef organisms has been recently demonstrated after their direct application [19]. Furthermore, this enrichment was only detected when the BMCs were also inherently found on the inoculated sponges, and all inoculated sponges remained healthy during the experiment. The potential off-target effects of *in situ*-applied BMCs on other reef organisms, especially those closely associated with corals, were never investigated. Herein, exploring coral probiotics’ effects on coral-associated organisms, such as fish, is an essential step for risk assessment for the implementation of microbial therapies in larger-scale applications [20], as well as for exploring novel interactions and potential benefits provided by such microbial therapies to other organisms following a coral reef “one health” approach [20,21].

Coral reef fish harbor species-specific bacterial communities, primarily in their skin, gills, and guts [22] which contribute to fish health, including defense against pathogens [23], tissue differentiation [24], and adaptation to environmental stressors [25]. In particular, the skin hosts a diverse microbiome that resides within the mucus coating the epithelial tissue. The skin microbiome plays crucial roles in antimicrobial activity against pathogens [26] and serves as an indicator of fish health [27]. The gut prokaryotes are essential for digesting and metabolizing compounds that fish cannot process independently [28], and they are pivotal in modulating the host’s immune system [29]. The gill microbiome also plays significant roles, such as participating in nutrient cycling by converting toxic ammonia produced by fish into less toxic nitrogen compounds, thus aiding in detoxification [30]. Additionally, the gill microbiome serves as a primary defense mechanism against pathogens, protecting the respiratory system, a major entry point for infections [31].

The bacterial communities of coral reef fish are shaped by multiple factors, including surrounding environmental microbes, fish diet, host genetics, and the health of local coral communities [32]. Additional environmental factors such as water quality and reef health are crucial for maintaining healthy fish microbiomes, which, when disrupted, may lead to dysbiotic states and consequent detrimental consequences for the fish’s health and functional role [33]. For instance, fish from degraded reefs exhibit less diverse gut microbiomes, potentially decreasing their disease resilience and nutrient absorption [34]. Given the interdependence between coral reefs and fish health, conservation strategies can greatly benefit from integrated approaches that address a core component connecting these organisms, often affected by environmental impacts: their microbiomes [35].

Here, we explore the interactions between coral probiotics and a common coral-associated damselfish, *Dascyllus abudafur* [36], following long-term *in situ* inoculation on healthy *Pocillopora verrucosa* colonies. Like many small reef fishes, *D. abudafur*, a member of the *Pomacentridae* family, associates with live coral colonies for shelter [37]. These fish exhibit a high degree of site fidelity, typically residing within and above the same coral colony for life [38]. However, bleaching and other disturbances to the coral host can result in increased predation rates, influence individuals to vacate host colonies and, ultimately, a decline in these fishes [39]. Leveraging this fidelity, we examined the probiotics’ effects on the bacterial communities of the fish’s skin, gills, and gut. The results indicate a tissue-specific microbiome response to the probiotic inoculations, with a decrease of potentially pathogenic bacteria in the skin microbiome and the enrichment of several other bacterial groups, mainly across the skin and gut tissues. These findings enhance our understanding of the ecological interactions of putative BMCs and non-target reef organisms, such as fish. Integrating these interactions into comprehensive coral reef conservation strategies is crucial and warrants future research to confirm the potentially beneficial effects on non-target reef-associated fish.

## Materials and methods

### Fish surveys and collection

**Figure 1:**
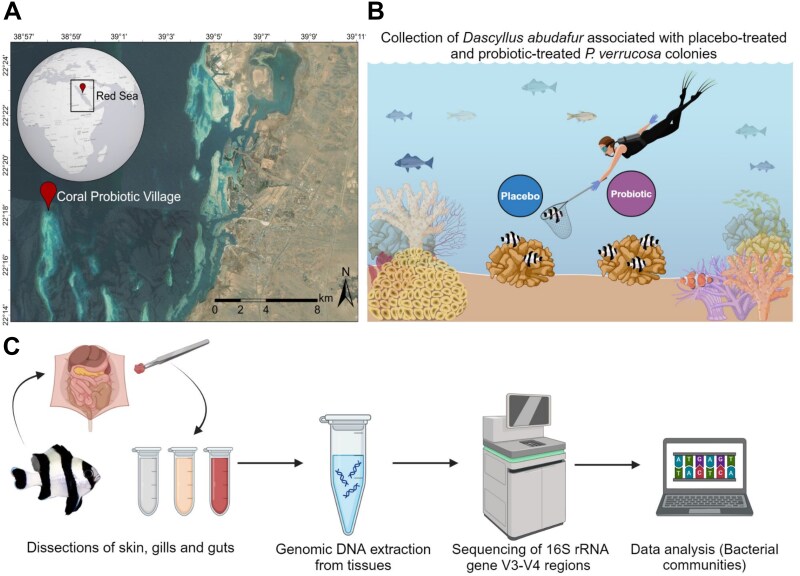
Experimental design workflow. A) the study location in the coral Probiotic Village (CPV) in Al Fahal reef, central Red Sea. The map was created with a licensed version of ArcGIS pro. Version 2.8.0. B) In situ collection of *Dascyllus abudafur* individuals from *Pocillopora verrucosa* colonies treated with placebo and probiotic (pBMCs) C) fish tissue dissection and subsequent experimental procedures, including DNA extraction and next generation sequencing (NGS) for bacterial community analyses. Infographics of panel B and C were created with a licensed version in Biorender.com.

This study was conducted at the “Red Sea Research Center Coral Probiotic Village,” located at the Al Fahal Reef (22°18′18.4”N; 38°57′52.5″E) in the Central Red Sea (Fig. 1A). The study area expands ~500 m^2^ area, with depths ranging from 8 to 10 meters. The experiment was performed concurrently with another major study to assess the effects of putative BMCs on *P. verrucosa* healthy colonies, as described by Delgadillo-Ordoñez and colleagues [18]. In brief, thirty visually healthy colonies of the brown morphotype of *P. verrucosa* were chosen for the study, with a minimum distance between colonies of three meters to minimize sampling of clonal genotypes. In addition, this distance was selected based on previous studies that show *D. abudafur* has extremely high site fidelity, typically spending its entire life less than one meter from its host coral colony [38]. Moreover, even if displaced, these fish remarkably tend to return to their home colonies [40]. Thus, this distance threshold was set as a very conservative parameter to minimize any potential mixing of the *D. abudafur* between neighboring coral colonies. These colonies, randomly assigned into control (referred to as placebo) and probiotic treatments (n = 15 per treatment), harbored individuals of *D. abudafur* fish populations that associate with live coral colonies of this species [36,38]. Probiotic-treated coral colonies were inoculated with a putative probiotic consortium (pBMCs) consisting of six bacterial strains isolated from healthy corals in the Central Red Sea and selected upon in vitro testing for their potential as probiotic bacteria, such as antioxidant activity, siderophores production, and phosphate assimilation [18]. This consortium was composed of the following members: two *Pseudoalteromonas galatheae* (strains 30H, accession number SAMEA114261728; and 33H, accession number SAMEA114261729), two *Cobetia amphilecti* (strains 65H accession number SAMEA114261730; and 81H accession number SAMEA114261731) isolated from *P. verrucosa*, one *Halomonas* sp. (strain SAT10 accession number SAMEA114261732) isolated from *Stylophora pistillata*, and one *Suctlifiella* sp. (strain SAA19 accession number SAMEA114261733) isolated from *Galaxea fascicularis*. Their pangenome assemblies are deposited under project number PRJEB62849 [41]. The probiotic inoculations were performed three times per week for 3 months (from August 2021 to November 2021) on the selected *P. verrucosa* colonies. To inoculate the probiotic consortium, we gently dispensed the bacterial suspension directly onto the seawater surrounding a few (5-10 cm) centimeters from each *P. verrucosa* colony using a sterile syringe. During the inoculation process, *D. abudafur* individuals inhabiting the coral colonies remained within or just above the coral branches, maintaining proximity to the inoculation site. While no active predation of the probiotic solution by these fish was observed, incidental ingestion of the bacterial suspension cannot be ruled out. This consideration is one of the reasons why the gut-associated microbiomes of the fish were also investigated in this study. This approach aimed to detect any potential uptake of the inoculated microbes through incidental ingestion or inherent contact with the coral surface.

In order to assess the impact of the inoculated probiotic consortium and to examine their tissue-associated bacterial communities of the skin, gills, and gut, fish specimens were collected from probiotic-treated and placebo-treated healthy coral colonies under KAUST IACUC Protocol 20IACUC05. All fish collected in this study were visually healthy, had sizes ranging from 25 to 67 mm, and did not present any visual deviations in normal swimming behavior or color. A total of 22 adult *D. abudafur* individuals were sampled: 10 before probiotic inoculations (August 2021, T1) and 12 after the 3-month inoculation period (November 2021, T2). Each fish was dissected to collect skin, gill, and gut tissues, yielding 64 tissue samples. These included 29 samples from T1 (skin: 10, gills: 10, gut: 9) and 35 from T2, corresponding to placebo (skin: 5, gills: 6, gut: 6) and probiotic (skin: 6, gills: 6, gut: 6) treatments.

Our primary comparison focused on T2 placebo vs. T2 probiotic samples, directly testing our hypothesis regarding the effects of probiotic-treated corals on the fish microbiome. Hence, T1 samples (referred as placebo) were used for describing the fish microbiome before any inoculation occurred, and only compared with T2 placebo samples to account for natural temporal changes. Fish individuals were captured using a small hand net and encouraged out of the shelter of the coral colony using fish food as bait, one-by-one and placed in a bag individually (Fig. 1B). This was to prevent any disturbance to the coral colonies and introduce any exogenous source of chemicals that could alter their microbiome, such as clove oil [42]. The fish individuals were dissected to separate the three tissues, followed by genomic DNA extraction and sequencing of the V3–V4 regions of the 16S rRNA gene to assess changes in the tissue-associated bacterial communities upon probiotic inoculations when compared with the placebo group (Fig. 1C).

### DNA extraction and sequencing

Tissue samples were thawed on ice, and DNA extraction was performed from each tissue type using the DNeasy® Blood and Tissue kit (Qiagen) following the manufacturer’s instructions. We additionally sequenced reagent-only extraction blanks (i.e. samples without any biological material) alongside our biological samples to detect potential contaminants introduced during reagents or laboratory handling. Furthermore, we sequenced the fish food used to attract the fish as bait to serve as an additional control. These blanks were processed under identical conditions (same kit, same number of steps, and same reagents) as our biological samples. The concentration of the extracted DNA was quantified using a high sensitivity Qubit™ dsDNA assay kit (Invitrogen™) and then shipped on dry ice for sequencing at Novogene Corporation-Inc in China. Sequencing of the V3-V4 regions of the 16S rRNA gene was performed using the universal primers 341F 5’ CCTACGGGNGGC WGCAG 3′ and 785R 5’ GAC TAC HVG GGT ATC TAA TCC 3′ for the genomic DNA. PCR mixtures contained 15 μL of Phusion® High-Fidelity PCR Master Mix (New England Biolabs), 0.2 μM of each forward and reverse primer and 10 ng of the samples’ genomic DNA. The thermal cycling conditions were as follows: a first denaturation step at 98°C for 1 minute, followed by 30 cycles at 98°C for 10 s, 50°C for 30 s, and 72°C for 30 s, and a final extension of 5 min at 72°C.

PCR products were verified and quantified by mixing their equal volume with 1X loading buffer (contained SYB green) and performing electrophoresis on 2% agarose gels. For the library preparation, PCR products were purified using a Qiagen Gel Extraction Kit (Qiagen, Germany). Sequencing libraries were generated with a NEBNext® Ultra™ II DNA Library Prep Kit (Cat No. E7645). The library quality was evaluated on a Qubit@ 2.0 Fluorometer (Thermo Scientific™) and Agilent Bioanalyzer 2100 system. Libraries were sequenced on a NovaSeq platform (Illumina), and 250 bp paired-end reads were generated. To account for potential contaminants, we included all our negative controls in our sequencing runs. All raw sequence reads were deposited in the European Nucleotide Archive (ENA) under the study accession number PRJEB75930.

### Bioinformatic processing

The DADA2 pipeline [43] was used to process the 16S rRNA gene-based amplicon libraries. In short, the raw reads were decontaminated by phiX and adapter-trimmed using the “BBDuk” tool from the BBMap suite (Bushnell B, http://sourceforge.net/projects/bbmap/). PCR primers were removed from the reads using the “cutadapt” tool [44]. To retain the maximum number of reads at the filterAndTrim step of DADA2, the maxEE (maximum expected error) parameter was set to 6 for the forward and reverse reads. After performing concatenation of the forward and reverse reads via the “justConcatenate” option in the *mergePairs* function of DADA2, the sequences were analyzed under the pseudo-pooling mode by following the standard DADA2 (version 1.22) workflow and by using the SILVA database, version 138.1 [45]. All potential contaminant ASVs identified by the decontam tool [46] implementing the prevalence-based method (on the default threshold setting) were removed from the analysis.

### Statistical analyses

Reads corresponding to mitochondria, chloroplast, archaea, eukaryotes, and singletons were removed, resulting in 157 068 Amplicon Sequence Variants (ASVs) from 105 samples. Alpha diversity, multidimensional ordination plots, and statistical comparisons were carried out in R version 4.2.2 using the functions in *Phyloseq* version 1.42.0 [47] and *Vegan* version 2.6–4 [48]. Rarefaction curves were generated for all samples (Supplementary Fig. 1) using the “rarecurve” function in *Vegan* for accounting for sequencing coverage, followed by rarefaction with a cutoff at 60.000 reads using the “rarefy_even_depth” function in *Vegan*. Alpha diversity was calculated using the “estimate_richness” function in *Phyloseq*, with the default diversity indices (observed number of ASVs, named here as number of species (S), Shannon H′, Simpson, and Chao1). Statistical comparisons between treatments (placebo and probiotic) for alpha diversity metrics were calculated by implementing the two-sided Wilcoxon test (previous testing for normal distribution of the data; Shapiro-Wilks, p-values <0.05, Supplementary Table 1). Non-metric Multidimensional Scaling (nMDS) ordinations were generated from Bray-Curtis distances from ASVs counts without performing any data transformation, using the “vegdist” and “metaMDS” functions in *Vegan*. The Permutational Multivariate Analysis of Variance (PERMANOVA) was used to test for overall significance, using “tissue,” “treatment,” and “sampling time” as factors, and then this was calculated for each tissue subset to test for significance over time, using “sampling time” as a factor, and between treatments in T2 using “treatment” as a factor (Supplementary Table 2). This was done by implementing the “adonis2” function in Vegan, from generated Bray–Curtis distances and 999 permutations. The homogeneity of variances was calculated for each tissue in T2 using the “betadisper” and “permutest” functions in *Vegan* using Bray-Curtis distances and 999 permutations (Betadisperse _(skin)_, df = 1, F = 51.259, Pr (>F) 0.001); (Betadisperse _(Gills)_, df = 1, F = 1.0752, Pr (>F) 0.343); (Betadisperse _(Gut)_, df = 1, F = 62.224, Pr (>F) 0.001). As PERMANOVA is largely unaffected by heterogeneity in balanced designs [49], we used it to calculate the statistical significance of the treatment for T2 samples (biological replicates per tissue: placebo: *n =* 6, 9 and 6; probiotic: *n =* 5, 6 and 6, for the skin, gills, and gut, respectively).

In addition, the ASV sequences were queried against the 16S sequences for each of the six inoculated pBMCs, using BLASTn [50] to identify them in the fish microbiome. From the original dataset (prior to the removal of singletons or performing any rarefaction), we identified seven ASVs that had >99% match across the entire amplicon to the V3–V4 region of the 16S rRNA gene in one of the pBMCs, corresponding to *Pseudoalteromonas galatheae* (n = 1) and *Halomonas* sp. (n = 6). From those, only five ASVs were retained after removing singletons and rarefaction of the data and corresponded to pBMC *Pseudoalteromonas galatheae* (n = 1) and *Halomonas* sp. (n = 4) (Supplementary Table 3).

The Analysis of the Composition of Microbiomes with Bias Correction 2 “ANCOM-BC2” [51] version 2.0 was used to identify differentially abundant ASV between the different treatments for each tissue. This method estimates unknown sampling fractions, corrects bias from sample differences, models absolute abundance with linear regression, and provides a statistically valid test with appropriate *p*-values, false discovery rate (FDR) control, and sustained power. We performed this analysis on total ASV counts (after removing singletons), using the Benjamini-Hochberg (BH) method to correct for false positives and an alpha of 0.05 for significance. An ASV was considered significant when it was enriched or decreased significantly (*P*_adj_ < .05) in the probiotic samples in comparison to the placebo (reference group) under the parameters above. We focused on and plotted the top most enriched (*P*_adj_ < .01, W statistic ranging >2) and most decreased (*P*_adj_ < .01, W statistic <2) ASVs in each tissue. The complete results of differentially abundant ASVs for each tissue (skin, gut, and gills) are available in Supplementary Tables 4–6, respectively.

Lastly, we assessed differentially abundant ASVs corresponding to potential fish pathogenic bacteria in each tissue and treatment. We used the ANCOM-BC2 results and selected the differentially abundant ASVs corresponding to bacterial genera encompassing well-known fish pathogens, which included: *Aeromonas salmonicida*, *Aeromonas hydrophila*, *Tenacibaculum maritimum*, *Vibrio harveyi*, *Vibrio vulfinicus*, *Vibrio parahaemolyticus*, *Vibrio alginolyticus*, *Vibrio anguillarum*, *Serratia marcescens*, *Photobacterium damselae*, *Lactococcus garvieae*, and *Lactococcus piscium* all known to cause diseases and pathogenesis in marine fish and aquaculture species. These analyses were conducted at the genus level since the study’s amplicon 16S rRNA data does not provide taxonomic resolution to the species level. The ASVs corresponding to the selected fish pathogens and their associated taxonomy are available in Supplementary Table 7. All plots were generated using ggplot2 version 3.4.0. Additional figures representing the experimental design were created in a licensed version of BioRender on Biorender.com.

## Results

**Figure 2:**
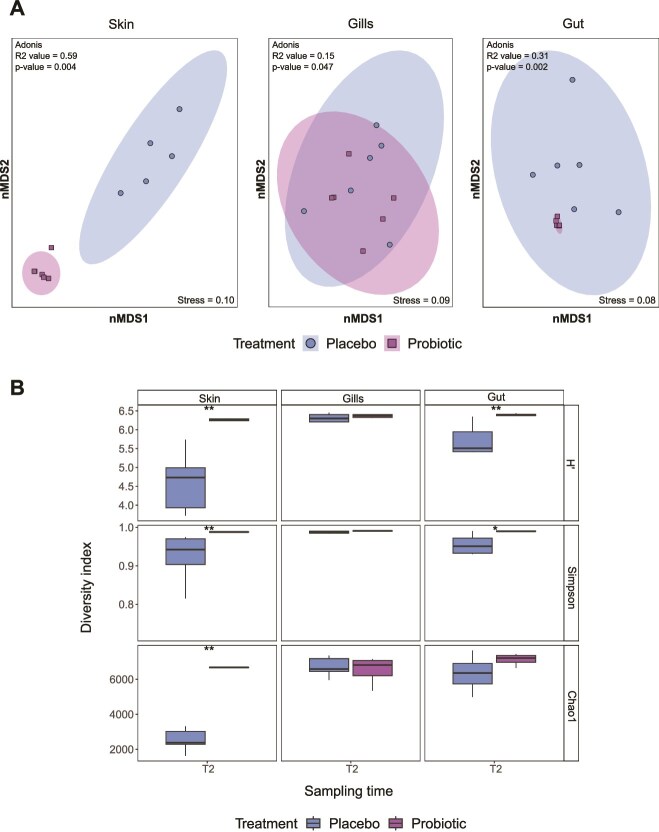
Compositional changes in the bacterial community of *Dascyllus abudafur* associated microbiomes with the *in situ* inoculation of coral probiotics after long-term *in situ* inoculation (T2). A) Nonmetric multidimensional scaling ordination (nMDS) of the microbial community associated with the skin, gills, and gut, according to sampling time (T2) and treatment (k = 2). B) Alpha diversity indices (H′ = Shannon-weaver diversity, Simpson and Chao1) estimated for each tissue (skin, gills, and gut) by treatment (placebo and probiotic) at T2. The statistically significant differences are denoted with asterisks: ^**^ = *P* < .01; ^***^ = *P* < .001.

Amplicon sequencing of the 16S rRNA gene showed that the long-term *in situ* probiotic inoculation (over three months) had a significant effect on the bacterial communities of *D. abudafur* associated with probiotic-treated colonies, in comparison to those associated with placebo-treated colonies (*Adonis*_(treatment)_, *R2* = 0.06, d*f* = 1, *F* = 5.13, *Pr_(>F)_* = 0.001). In particular, the bacterial communities of the skin (*Adonis*_(treatment)_, *R2* = 0.59, d*f* = 1, *F* = 12.95, *Pr_(>F)_* = 0.004) and gut (*Adonis*_(treatment)_, *R2* = 0.31, d*f* = 1, *F* = 4.46, *Pr_(>F)_* = 0.002) tissues were significantly different. Conversely, the bacterial community associated with gill tissue was more homogeneous across treatments but still showed significant differences between them (*Adonis*_*(*treatment)_, *R2* = 0.15, d*f* = 1, *F* = 1.79, *Pr_(>F)_* = 0.047) (Fig. 2A) (Supplementary Table 2). Furthermore, the skin and gut microbiomes of fish from the probiotic treatment displayed tightly clustered nMDS plots, indicating low variation among replicates and a more uniform overall microbiome structure (Fig. 2A).

Alpha diversity metrics were significantly higher in the skin (Shannon (H′), Simpson, and Chao1) (*Wilcox*, *p values* = 0.0043) and the gut (Shannon (H′) and Simpson) (*Wilcox, p values* = 0.0043 and 0.026, respectively) microbiome in fish associated with probiotic-treated colonies, compared to placebo-treated corals in T2 (Fig. 2B). On the contrary, the gill tissue did not exhibit significant differences in alpha diversity metrics between treatments (*Wilcox*, *p values* > 0.05) (Fig. 2B). A summary of Alpha diversity metrics is available in Supplementary Table 1.

We also identified the ASVs matching >99% similarity with the 16S rRNA genes of inoculated pBMCs in the tissue microbiomes (Supplementary Table 3). We identified five ASVs corresponding to pBMCs *Pseudoalteromonas galatheae* (n = 1) and *Halomonas* sp. (n = 4), which comprised 0.018% ± 0.001 and 0.003% ± 0.0004 of the fish microbiome, respectively. However, they were not significantly enriched in any of the tissues of fish associated with probiotic-treated corals compared to those associated with placebo-treated corals (*Wilcox*, *p values* > 0.05). In addition, no ASVs corresponding to pBMC *Sutcliffiella* (or *Bacillus*, the former classification of this genus) and *Cobetia amphilecti* were detected at any sampling time.

Overall, the three tissues harbored significantly different bacterial communities (*Adonis*_(tissue)_, *R2* = 0.10, d*f* = 2, *F* = 4.15, *Pr_(>F)_* = 0.001) that changed over time (*Adonis*_(skin-sampling time)_, *R2* = 0.17, d*f* = 1, *F* = 4.75, *Pr_(>F)_* = 0.002), (*Adonis*_(gills-sampling time)_, *R2* = 0.20, d*f* = 1, *F* = 4.83, *Pr_(>F)_* = 0.001), (*Adonis*_(gut-sampling time)_, *R2* = 0.18, d*f* = 1, *F* = 4.28, *Pr_(>F)_* = 0.004) (Supplementary Fig. 2A). In addition, the bacterial community of *D. abudafur* (from placebo samples at T1 and T2 combined) was dominated by the Phylum Proteobacteria (41.7% ± 17.9), followed by Firmicutes (31.35% ± 15.06), Actinobacteriota (8.14% ± 8.24), Bacteroidota (5.42% ± 8.60) and Cyanobacteria (4.11% ± 8.89). The most abundant families comprised *Pseudomonadaceae* (9.53% ± 8.18), followed by *Burkholderiacae* (6.54% ± 13.50), *Lachnospiraceae* (5.92% ± 4.36), *Bacillaceae* (5.41% ± 9.72), and *Selenomonadaceae* (4.09% ± 5.03), among others (Supplementary Fig. 2B).

**Figure 3:**
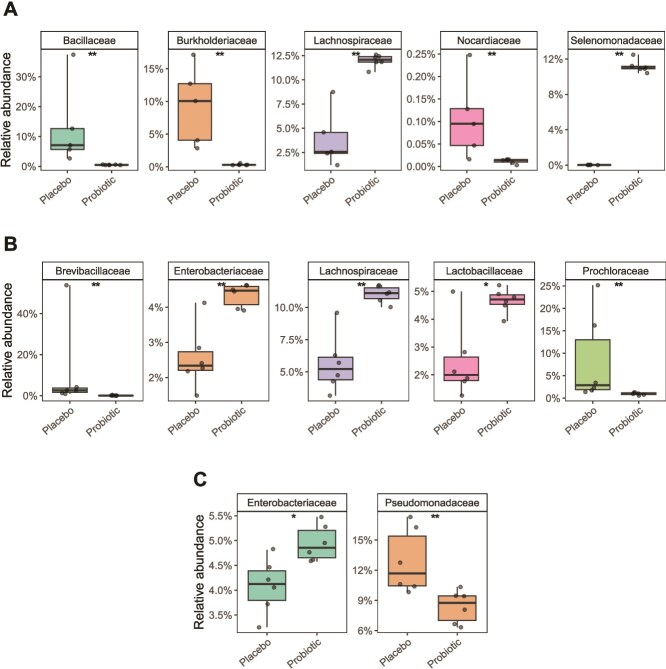
Compositional changes in the top 10 most abundant bacterial families of *Dascyllus abudafur* tissue-associated microbiomes after a long-term *in situ* probiotic inoculation (T2 sampling time). Families with significant changes in the relative abundance between fish affiliated with placebo- and probiotic-treated corals in the A) skin, B) gut, and C) gill tissue. The statistically significant differences are denoted with asterisks: ^**^ = *P* < .01; ^***^ = *P* < .001. The depicted boxplots show the median (center line) and the first and third quartiles (lower and upper bounds). The gray dots represent biological replicates.

To further explore the compositional differences, we assessed changes in the most abundant bacterial families between treatments at T2 in each tissue (Fig. 3). We noticed significant changes in the relative abundance of several groups among the top 10 most abundant families between fish affiliated with probiotic-treated and placebo-treated corals at T2. In the skin microbiome, the families *Lachnospiraceae* and *Selenomonadaceae* increased by 10% in relative abundance in the probiotic treatment (Wilcox, *p values* = 0.0043 and 0.008, respectively). In contrast, the families *Bacillaceae*, *Burkholderiaceae,* and *Nocardiaceae* were significantly decreased (Wilcox, *p values* = 0.0043, 0.008, and 0.0075, respectively) (Fig. 3A). The gut tissue presented significant enrichment in the families *Enterobacteriaceae*, *Lachnospiraceae*, and *Lactobacillaceae* higher than twofold in relative abundance (Wilcox, *p values* = 0.0087, 0.0022, and 0.041, respectively). Conversely, significantly decreased families in the gut included *Brevibacillaceae* and *Prochloraceae* (Wilcox, *p values* = 0.0022, respectively) (Fig. 3B). The gills were significantly enriched with *Enterobacteriaceae* and presented a significant decrease in the family *Pseudomonadaceae* (Wilcox, *p values* = 0.015 and 0.0043, respectively) (Fig. 3C).

**Figure 4:**
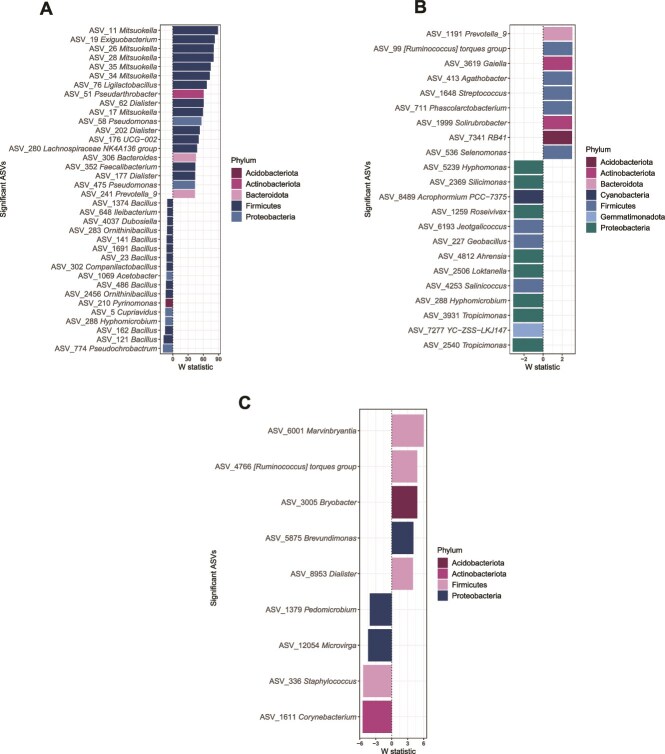
Differentially abundant bacteria in *Dascyllus abudafur* tissues associated with probiotic-treated in comparison to the placebo-treated *Pocillopora verrucosa* colonies. Top differentially abundant ASVs identified in the ANCOM-BC2 analysis in A) skin, B) gut, and C) gill tissues. The associated genus taxa are color-coded by phylum. The W test statistic from the ANCOM-BC2 is shown (negative W indicates decreased taxa while positive W indicates enriched taxa in probiotic-treated fish compared to placebo-treated fish individuals). “Unclassified” ASVs are available in Supplementary Tables 4-6.

Moreover, we assessed the differentially abundant ASVs between fish affiliated with probiotic-treated and placebo-treated colonies at sampling time T2 for each tissue. Results revealed several differentially abundant ASVs in the skin (ANCOM-BC2, *n* = 2224 ASVs, *P*_adj_ < .05) that were enriched (*n* = 731) and decreased (*n* = 1493) in the fish collected from probiotic-treated colonies in comparison to the reference group (placebo). The most enriched ASVs (*P*_adj_ < .01, *W* statistic >10) were mainly affiliated with the phylum *Firmicutes* (*n* = 14) and belonged to the genus *Mitsuokella* (family *Selenomonadaceae*) (*n* = 6), *Dialister* (*n* = 3), *Exiguobacterium* (*n* = 1), *Ligilactobacillus* (*n* = 1), UCG-002 (*n* = 1), *Lachnospiraceae* NK4A136 group (*n* = 1), and *Faecalibacterium* (*n* = 1). Similarly, the most decreased ASVs (*P*_adj_ < .01, *W* statistic < −10) were mostly affiliated with Firmicutes (*n =* 12) and belonged to the genus *Bacillus* (*n* = 7), *Ornithinibacillus* (*n* = 2), *Ileibacterium* (*n* = 1), *Dubosiella* (*n* = 1), and *Companilactobacillus* (*n* = 1). Other enriched and decreased ASVs belong to several phyla, including Acidobacteriota, Actinobacteriota, Bacteroidota, and Proteobacteria (Fig. 4A**) (**Supplementary Table 4).

The gut bacterial community also presented a high number of differentially abundant ASVs (ANCOM-BC2, *n* = 562 ASVs, *P*_adj_ < .05) that were enriched (*n* = 415) or decreased (*n* = 147) between fish affiliated with probiotic-treated and placebo-treated colonies. Most enriched ASVs (*P*_adj_ < .01, *W* statistic >3) belonged to *Firmicutes* (*n* = 5), including genera such as *Agathobacter* (*n* = 1), *Streptococcus* (*n* = 1), *Phascolarctobacterium* (*n* = 1), *Selenomonas* (*n* = 1) and *Ruminococcus torques* group (*n* = 1). Conversely, most decreased ASVs were mainly affiliated with Proteobacteria (*n =* 8), including the genera *Tropicimonas* (*n* = 2), *Hyphomonmas* (*n* = 1), *Silicimonas* (*n* = 1), *Roseivivax* (*n* = 1), *Ahrensia* (*n* = 1), *Loktanella* (*n* = 1), and *Hyphomicrobium* (*n* = 1). Some members of the Firmicutes phylum were also decreased, including *Jeotgalicoccus* (*n* = 1), *Geobacillus* (*n* = 1), and *Salinicoccus* (*n* = 1) (Supplementary Table 5). Notably, the changes associated with the differentially abundant ASVs in the gut bacterial community were less pronounced than those observed in the skin, as denoted by the smaller W values (Fig. 4B).

In contrast to the observed changes in the skin and gut bacterial communities, the gills exhibited only a few differentially abundant ASVs (ANCOM-BC2, *n* = 16 ASVs, *P*_adj_ < .05) that were enriched (*n* = 8) and decreased (*n* = 8) between treatments. The most enriched ASVs (*P*_adj_ < .01, *W* statistic >3) belonged to Firmicutes (*n =* 3), including the genera *Marvinbryantia* (*n* = 1), *R. torques* group (*n* = 1), and *Dialister* (*n* = 1). Other enriched ASVs included representatives of Acidobacteriota (i.e. *Bryobacter*, n = 1) and Proteobacteria (i.e. *Brevundimonas*, n = 1). Conversely, most decreased ASVs (*P*_adj_ < .01, *W* statistic < −3) belonged to Proteobacteria, including two genera (*Pedomicrobium*, n = 1; *Microvirga*, n = 1) as well as Firmicutes (*Staphylococcus n* = 1) and Actinobacteriota (*Corynebacterium*, n = 1) (Fig. 4C) (Supplementary Table 6). Unclassified groups among the differentially abundant ASVs for each tissue are available in their respective supplementary tables.

**Figure 5:**
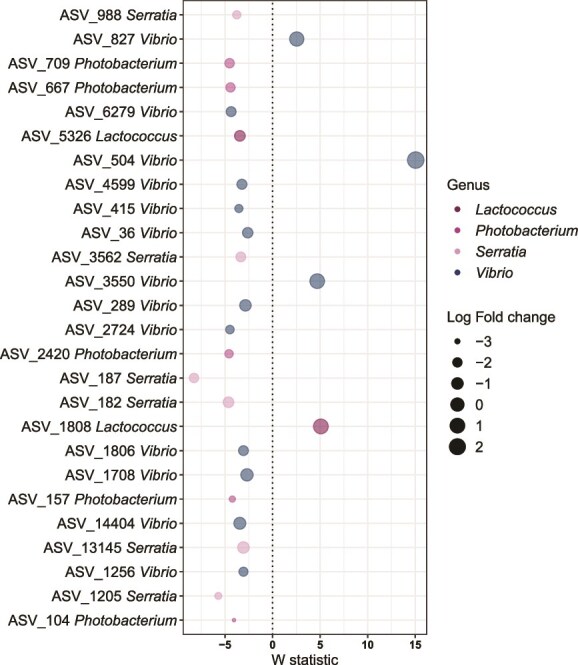
Differentially abundant ASVs of the skin microbiome corresponding to genera affiliated with known putative fish pathogenic bacteria, in probiotic-treated fish compared to placebo-treated fish individuals in sampling time T2. The ASVs taxa are color-coded by genus. The W test statistic from the ANCOM-BC2 is shown (negative W indicates decreased taxa while positive W indicates enriched taxa in probiotic-treated fish compared to placebo-treated fish individuals). The dot size represents the log fold change of each ASV.

In addition, we evaluated changes in differentially abundant ASVs (ANCOM-BC2, *P*_adj_ < .05) affiliated with several potentially fish pathogenic bacteria genera, including *Vibrio*, *Lactococcus*, *Photobacterium*, and *Serratia*, for each tissue. Interestingly, the skin microbiome showed a higher number of significantly decreased ASVs (*n =* 22, *W* < −2) in contrast to ASVs that were significantly enriched (*n =* 4, *W* > 2) in probiotic-treated compared to the placebo-treated associated fish (Fig. 5). The decreased ASVs mainly belonged to *Vibrio* (*n =* 10), followed by *Serratia* (*n =* 6), *Photobacterium* (*n =* 5), and *Lactococcus* (*n =* 1), while most significantly enriched ASVs belonged to *Vibrio* (*n =* 3), and *Lactococcus* (*n =* 1) (Supplementary table 7). Contrastingly, the gills did not present any differentially abundant ASV affiliated with those potential bacterial pathogens, whereas the gut tissue only showed enrichment of one single ASV belonging to *Vibrio* (Supplementary Table 7).

## Discussion

Our results indicate that the skin and gut tissues of *D. abudafur* exhibited significant changes in the composition of their associated bacterial communities after probiotic inoculations in corals. This evidence supports tissue-specific microbiome restructuring triggered by the *in situ* application of pBMCs on corals, highlighting the importance of studying different fish compartments as they potentially display differential responses to microbial therapies. Our findings align with other studies that employed fish probiotics, leading to significant skin and gut microbiome changes, enhancing the host immune responses to potential pathogens and disease tolerance [52,53].

Conversely, the gill microbiome of *D. abudafur* exhibited significant changes, though less pronounced compared to the skin and gut microbiomes across treatments. This aligns with previous studies that describe higher stability in gill microbiomes of infected Atlantic salmon with the fish pathogen *Piscirickettsia salmonis* [54]. Also, a study by Rosado et al., 2019 reported a more stable gill microbiome compared to the skin tissue, with lower variation in the mucosal microbiome composition of seabass fish (family *Serranidae*) during and post-infection outbreaks [55]. In addition, similar findings in a study by Liao and colleagues indicated that the gill microbiome of marine fish medaka (*Oryzias melastigma*) was remarkably more stable under exposure to microplastics and tetracycline than the gut microbiome [56]. This consistent trend in gill microbiomes from various fish species highlights apparent stability, even under environmental stressors.

When focusing on *D. abudafur* microbiome composition, we found a significant enrichment and decrease of some dominant families in each tissue at sampling time T2, when comparing fish affiliated with placebo-treated and probiotic-treated corals. For instance, the skin-enriched families included *Lachnospiraceae* and *Selenomonadaceae*, which include bacteria that are known for their positive effects on the host’s metabolic health. *Lachnospiraceae* are anaerobic, fermentative, and chemoorganotrophic bacteria that contribute to the production of short-chain fatty acids that modulate the host’s inflammatory processes and provide energy resources [57]. In addition, bacterial members of *Selenomonadaceae* are associated with carbohydrates [58] and amino acid metabolism [59]. Moreover, some of their members can produce butyrate from lactate and acetate, which is an essential short-chain fatty acid in the intestine [58]. In contrast, there was a significant decrease in the families *Bacillaceae*, *Burkholderiaceae*, and *Nocardiaceae*, which contain some fish pathogens [60,61]. Although these observations are consistent with the hypothesis of a potentially beneficial microbiome restructuring, further studies could be performed to validate that such taxonomic shifts would, in fact, result in improved disease resistance or nutritional benefits for the host, by integrating functional metagenomics, metabolite profiling, and metrics of fish health and behavior.

The gut microbiome had a significant enrichment of the families *Enterobacteriaceae*, *Lachnospiraceae*, and *Lactobacillaceae*. *Enterobacteriaceae* members are used as fish probiotics [62] and are known to improve fish nutrition and disease resistance by enhancing immune response mechanisms [63], yet some members can also be pathogenic [64]. Some members of *Lachnospiraceae* are beneficial bacteria in the gut, well known for the production of butyric acid, an essential substance for promoting microbial and host epithelial cell growth [65], which reduces intestinal inflammation in aquatic animals [66]. As for *Lactobacillaceae*, they are known for their role in improving the gut morphology in eels [67] and some marine crustaceans [68] by increasing villi height and microvilli density and enhancing the gut’s health. Moreover, they also enhance the fish’s immune response, as observed in seabass (*Dicentrarchus labrax*) [69] and tilapia fish (*Oreochromis niloticus*) [70]. Finally, they also play important roles in controlling pathogens [71] and modulating the fish’s microbiota [72]. Conversely, decreased families included *Brevibacillaceae*, which may indicate a reduction in potential pathogenic factors to the fish, as some bacteria from this family have been shown to produce extracellular proteases with significant toxic activity [73], and toxic effects have been demonstrated in water-dwelling invertebrates [74].

The gill microbiome showed an increase in *Enterobacteriaceae*, a family known for its probiotic role in fish and control of fish pathogens [75]. Also, there was an observable reduction in *Pseudomonadaceae* in the gill, indicating a decrease in potential pathogens for fish [76]. These observed shifts in microbial composition suggest that the coral probiotic treatment induced tissue-specific shifts among the most dominant bacterial families, from which some contain potentially beneficial groups that may contribute to the fish’s host health.

Moreover, when assessing differentially abundant ASVs, we observed an enrichment of several bacterial taxa, particularly from the Phylum Firmicutes across all tissues, from fish associated with probiotic-treated coral colonies. Notably, the skin bacterial community was highly enriched with bacteria from the genus *Mitsuokella* (family *Selenomonadaceae*). Bacteria from this genus have been observed to inhibit the growth of pathogens such as *Salmonella* in the digestive tract of land animals [77] and display potential probiotic activity by increasing the abundance and diversity of beneficial bacteria, contributing to gut health [78]. In fish, it has been identified in the digestive systems of tuna (e.g. *Thunnus albacares* and *Thunnus obesus*) and is correlated with nutrient and nucleotide metabolism [79]. Further research is warranted to elucidate their roles in coral reef fish, particularly in the skin microbiome, as they may play similar beneficial roles (e.g. antagonism against pathogens and microbiome modulation). Other skin-enriched bacterial taxa included bacteria from the genus *Exiguobacterium,* which encompass pollutant-tolerant bacteria, as they were found to have a protective activity against arsenic-induced oxidative damage and toxicity in freshwater fish exposed to arsenic contamination [80]. Other studies found probiotic effects of this bacteria in the freshwater goldfish (*Carassius auratus*), improving its growth and immune responses and enhancing fish survival after exposure to the bacterial pathogen *A. hydrophila* [81]. In this sense, it has been proposed as a potential probiotic for shrimp farms, as they improved the growth rates and body weight of the Pacific white shrimp (*Litopenaeus vannamei*) [82].

Furthermore, the gut microbiome was enriched with several fermentative genera from Firmicutes, including *Ruminococcus* and *Streptococcus.* Bacteria from the *Ruminococcus* have been used as probiotics for fish, effectively improving the immune response, antioxidant levels, and growth profiles of the Nile tilapia (*O. niloticus*) [83]. *Streptococcus* also includes bacteria with distinct roles in fish gut microbiomes, both beneficial [84] and pathogenic [85]. It contains lactic acid-producing bacteria with prominent probiotic activity in the human gut [86] and supports the fish gut microbiome by degrading complex carbohydrates (e.g. chitin, starch, cellulose) derived from fish diets [87]. In addition, they can produce antimicrobial compounds as novel sources of antibiotics [88]. Other gut-associated enriched taxa included *Agathobacter*, a butyrate-producing bacteria that play roles in gut-microbiota health, modulating host-immune response [89]. In fish, they were found to correlate with the biosynthesis of antibiotics and anti-inflammatory compounds and the immune response in salmonid fish [90]*.* In addition, *Selenemonas*, which was also enriched, is a propionate-producing bacteria, highly abundant in rumen-bacterial communities, that enhances the fiber fermentation of rumen when in cooperation with other fibrolytic bacteria such as *Ruminococcus* and *Fibrobacter* [91]. Their putative roles in the fish gut microbiome are yet to be elucidated, and these could be related to degrading fiber material derived from a fish-herbivorous diet.

Conversely, significantly decreased bacteria in the skin microbiome of fish associated with probiotic-treated coral colonies included several ASVs belonging to the genus *Bacillus*. This genus encompasses a wide range of beneficial bacteria across several organisms [92] and opportunistic pathogens [93]. Some of its members have been associated with probiotic activity in fish for aquaculture [94]. However, their roles and dynamics in coral reef fish are widely unexplored. Further studies could elucidate their specific roles in the skin microbiomes of fish. As for the gills, some decreased ASVs were particularly associated with genera encompassing known pathogens such as *Corynebacterium* and *Staphylococcus.* For instance, some members of *Corynebacterium* have been found as causative agents of fish disease [95], and they also contain coral pathogens associated with the black band disease [96]. *Staphylococcus* members have been found to cause eye protrusion and mortality of rainbow trout fish [97] and accumulate in tissues of different fish species exposed to poor water quality [98]. Lastly, decreased ASVs in the gut mostly belonged to Proteobacteria, namely to several genera, including taxa that have been found in gut tracts of white shrimp (*L. vannamei*) [99], as well as the skin microbiome of snook (*Centropomus undecimalis*) [100]*.* Others included *Hyphomicrobium*, which was found as a bacterial indicator of fish infected with the fish ectoparasite *Argulus siamensis*, causing gut-microbiome dysbiosis in freshwater fish [101]. Hence, its decrease in the gut microbiome of *D. abudafur* may pose a beneficial effect in fish associated with probiotic-treated corals, requiring further investigation.

Moreover, we detected two of the inoculated pBMCs inherently present in the fish microbiome (*Halomonas* spp., *Pseudoalteromonas galatheae*) in very low abundance (< 0.01%). However, we did not observe a significant enrichment of such ASVs in the studied fish tissues at sampling time T2 after the long-term inoculation. Similar results were observed in the *P. verrucosa* microbiome, where the same inoculated pBMCs were found in very low abundance in these corals (< 0.1%) after three months of probiotic inoculations. In that study, a significant enrichment of three of the inoculated BMC genera was observed (i.e. *Halomonas*, *Pseudoalteromonas*, *Bacillus*) when probiotics were applied to corals *in situ* [18], evidencing enrichment of the probiotic genera upon long-term *in situ* inoculations. In this sense, even if the probiotic strains do not persist in high abundance over the long term, the probiotic treatment can still reshape the microbiome, resulting in a potentially more beneficial microbial community [102]. Hence, continuous probiotic inoculations can instigate the enrichment of beneficial bacteria and microbiome restructuring, which may promote beneficial effects for the host.

Our results also indicate a clear trend of significant reductions in bacterial genera affiliated with potential fish pathogens, including *Vibrio*, *Photobacterium*, *Serratia*, and *Lactococcus* in fish from probiotic-treated corals. Some members of these bacterial taxa have been extensively documented to cause several diseases in a wide range of marine and freshwater fish species [103–105]. In particular, bacteria from the genus *Photobacterium* (*P. damselae*) can affect damselfish [106] among other fish groups. The observed reduction of potentially pathogenic bacteria in the skin microbiome of fish associated with probiotic-treated coral colonies aligns with the results obtained by Delgadillo-Ordoñez and colleagues, who also reported a decrease in similar pathogenic genera, such as *Vibrio* and *Photobacterium*, in probiotic-treated corals [18]. Genera such as *Pseudoalteromonas* used in these coral probiotic assemblages [15,18] are also effective as fish probiotics [52], demonstrating their broader potential in enhancing the health of various reef organisms. Although our findings indicate that coral probiotics significantly reduce potential pathogens in coral reef fish residing within probiotic-treated coral colonies, we ground our discussion on bacterial genera or known species commonly associated with fish disease in the literature. Despite that, confirmation of pathogenicity at the species or strain level would need further genomic or functional characterization on specific groups, which was out of the scope of this study. Although we cannot completely rule out potential unintended consequences of such probiotic-induced microbiome changes in coral reef fish and indirect ecological effects, the reduction in the abundance of pathogens and the enrichment of putative beneficial bacteria is a first and essential step for environmental restoration [35] and against microbialization, the process where the degraded marine ecosystem is enriched with microbial pathogens and dysbiotic features [107]. Nonetheless, we highlight that these interpretations remain putative and require direct measurements of fish health parameters and controlled experimental validation.

The novel and somehow unintended effects observed towards a putative beneficial microbiome restructuring underscore the importance of further research to explore the interactions between *in situ* applications of coral probiotics and their potential benefits on non-targeted organisms, potentially broadening the applications of BMCs across marine organisms. Importantly, our findings align with previous research showing that applying pBMCs into coral ecosystems positively reshapes the coral-associated microbiome without adverse effects on the microbial communities of nearby seawater and sediment, suggesting the safe application of pBMCs in reef ecosystems [18]. pBMCs were also enriched in sponges when these bacteria were already found in association with sponges, which remained healthy during the entire experiment [19]. These promising results are also a consequence of the strict selection of pBMCs, which follows a risk assessment framework that excludes any known pathogens and prioritizes the use of common, local, and widespread marine beneficial bacteria [11,20]. It is also important to note that the strains used in this study were all isolated from healthy corals from the same reef where the study was conducted in the Red Sea and carefully screened and selected based on their putative beneficial traits [18,41]. The use of native pBMCs that are commonly found in the marine ecosystem across different marine hosts and are not associated with pathogenic status is one of the risk assessment steps proposed for the use of microbial therapies to prevent biodiversity loss [20]. Also, no visual detrimental effects were observed on the coral colonies or among the studied fish populations. Future research could expand to other fish species and reef-associated taxa to better understand BMCs interactions across multiple reef organisms. Here, we provide compelling evidence that, when applied *in situ,* coral probiotics reshape fish-associated microbiomes in a tissue-specific manner, enriching putatively beneficial bacterial taxa while decreasing potential pathogens, as observed in corals and sponges [18,19].

When possible and relevant, future studies could also benefit from incorporating complementary culture-based methods to isolate inoculated probiotic strains within non-targeted hosts. While our 16S rRNA gene sequencing detected some inoculated pBMC genera at very low abundances (<0.01%), culturing these strains directly from fish tissues could provide further insights into their presence and potential establishment within the fish microbiome. However, this approach alone does not offer a definitive answer regarding probiotic uptake and subsequent effects on the host, as probiotic strains may naturally occur within the host-associated microbiome. To address this, isolating probiotic strains should be complemented by molecular-based approaches, such as full-length 16S rRNA, metagenomes, or other marker gene sequencing. Additionally, pBMCs may still induce microbiome restructuring, even if these probiotic strains are not detected in high abundance or are not established within the host microbiome. For instance, previous *in situ* probiotic inoculations in corals demonstrated that, although the same strains did not become dominant or relatively abundant, they still triggered potentially beneficial microbiome restructuring [18]. Thus, beneficial microbes may exert positive effects even if they are not dominant or persist in large numbers within the host. As extensively reviewed in a previous study [102], probiotics can act transiently by altering environmental or host conditions, e.g. acting in pathogen exclusion or enhancement of beneficial taxa.

Our results indicate that successive probiotic inoculations in corals induce microbiome shifts associated with nearby fish. This evidence supports the recently proposed “One Health” [21] concept, which posits that all organisms and ecosystems are interconnected. Consequently, promoting healthy coral microbiomes could not only support coral health but also the associated reef organisms and the ecosystem’s resilience to withstand climate change [20]. Hence, employing synergistic BMC applications customized to improve the health of multiple reef organisms offers a promising approach aligned with the principles of the “One Health” concept and microbiome stewardship [20].

The novel evidence we provide, indicating that pBMCs can restructure the microbiome of fish associated with probiotic-treated coral colonies, indicates potential beneficial effects of the inoculation of pBMCs for fish-associated communities since there was a significant increase in putative beneficial bacteria and a decrease in potential bacterial pathogens in fish associated with probiotics-treated colonies, which may point towards beneficial outcomes for the fish host. Furthermore, this work represents a significant advancement in coral probiotics studies and their potential ecological implications on non-target reef organisms by indicating potentially beneficial microbiome restructuring in coral-associated fish triggered by coral probiotics inoculations *in situ*. Future studies could expand this to other non-target organisms (e.g. benthic communities; coral reef cryptofauna), which would provide a better understanding of the ecological interactions associated with coral probiotics applications across different reef taxa at various environmental scales.

## Supplementary Material

Supplementary_figures_18_march_25_ycaf052

Supplementary_tables_18_March_25_ycaf052

## Data Availability

All raw sequence reads from the study were deposited in the European Nucleotide Archive (ENA) under the study accession number PRJEB75930. Other data supporting the results of this study are provided as Supplementary information files. Large data sets and R scripts for data analysis are available in the Zenodo repository at: 10.5281/zenodo.14850479.
