# Supplementary material for: Coral probiotics induce tissue-specific and putative beneficial microbiome restructuring in a coral-dwelling fish
Source: ISME Commun. 2025 Mar 22;5(1):ycaf052. doi: 10.1093/ismeco/ycaf052 (PMC11994995; doi:10.1093/ismeco/ycaf052)
Supplement: Supplementary_figures_18_march_25_ycaf052 [file supplementary_figures_18_march_25_ycaf052.docx]

*Coral Probiotics Reshape Fish Microbiome*

Supplemental Material of the manuscript: “Coral probiotics induce tissue-specific and putative beneficial microbiome restructuring in a coral-dwelling fish”

Joao Gabriel Duarte Rosado^1+^, Nathalia Delgadillo-Ordoñez^1+^, Matteo Monti^1^, Viktor Nunes Peinemann^1^ , Chakkiath Paul Antony^1^, Ahmed Alsaggaf^1^,

Inês Raimundo^1^, Darren Coker^1^, Neus Garcias-Bonet^1^, Francisca García^1^,

Raquel Silva Peixoto^1^, Susana Carvalho^1*^, Michael L. Berumen^1*^

^1^Biological and Environmental Science and Engineering Division (BESE), King Abdullah University of Science and Technology (KAUST), Thuwal, Saudi Arabia

+ These authors have contributed equally to this work.

* Corresponding authors: Susana Carvalho and Michael Berumen

Susana Carvalho, Biological and Environmental Science and Engineering Division (BESE), King Abdullah University of Science and Technology (KAUST), 4700 KAUST Building 2 Level 3, Thuwal, Makkah, 23955-6900, Kingdom of Saudi Arabia

Michael L. Berumen, Biological and Environmental Science and Engineering Division (BESE), King Abdullah University of Science and Technology (KAUST), 4700 KAUST Building 2 Level 3, Thuwal, Makkah, 23955-6900, Kingdom of Saudi Arabia


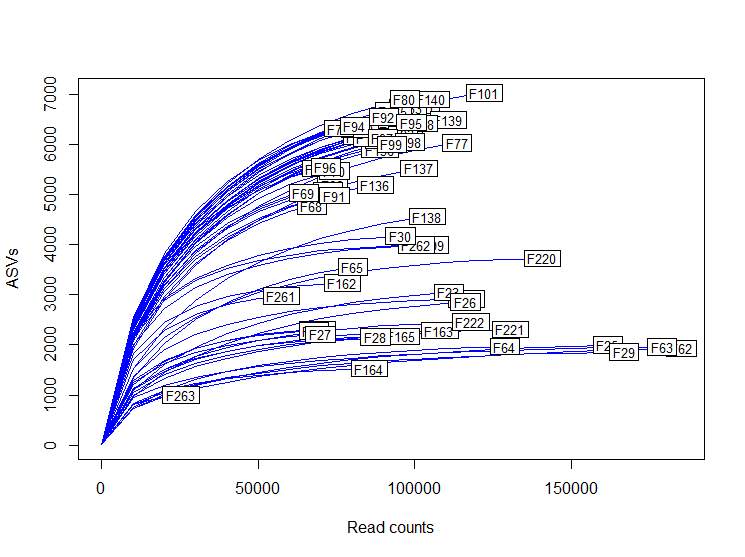


**Supplementary Figure 1.** Rarefaction curves showing the number of reads from the 16S rRNA sequencing data of the *Dascyllus abudafur* bacterial community. The X-axis shows the read counts and the Y-axis depicts the number of ASVs.


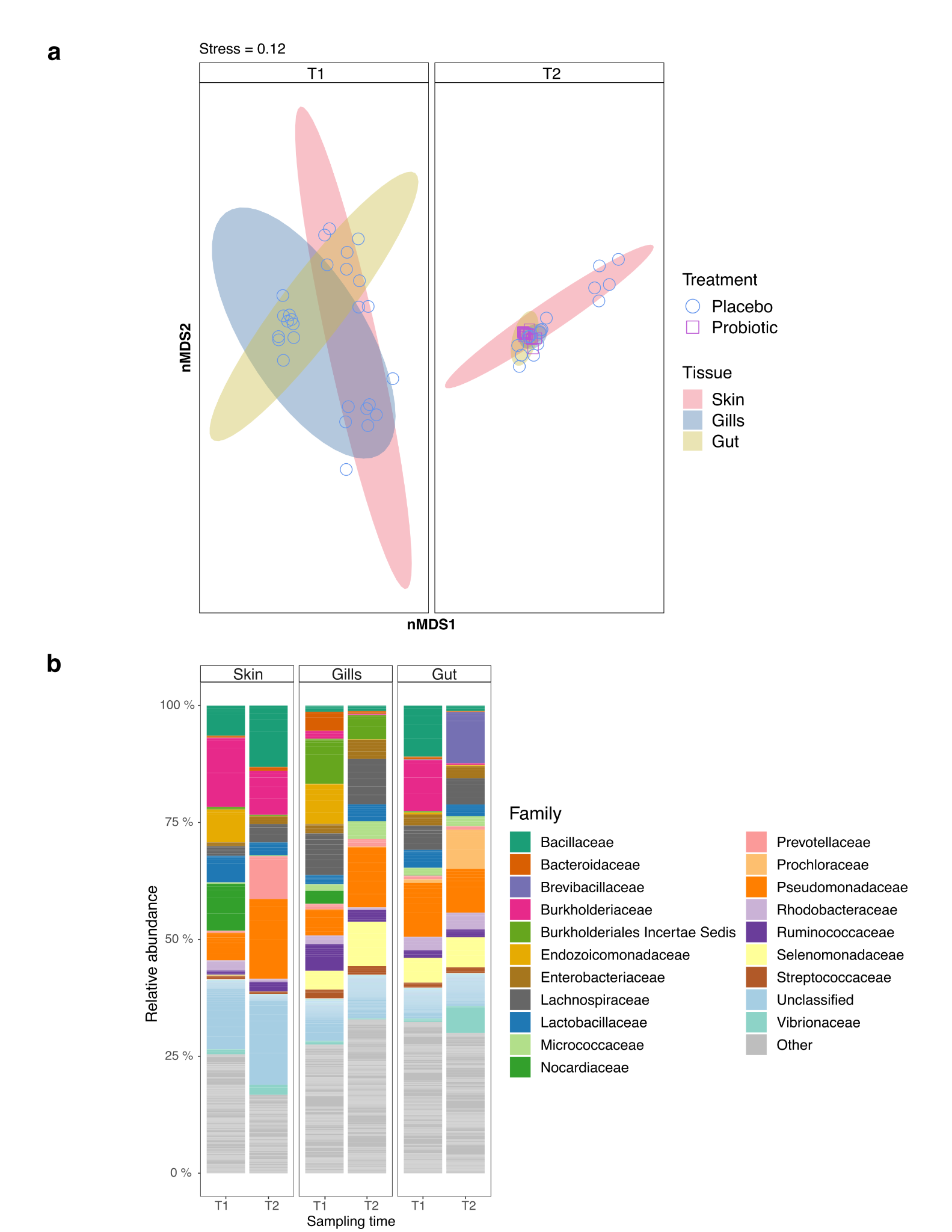


**Supplementary Figure 2.** Bacterial community structure and composition of most abundant families of *Dascyllus abudafur*, for each tissue (skin, gills and gut). a) Nonmetric multidimensional scaling ordination (nMDS) showing the bacterial community structure of the skin, gills and gut of *Dascyllus abudafur*, by treatment and sampling time (T1 and T2) (*k = 2*). b) Barplots depicting the top 10 most abundant bacterial families in T1 and T2 of *Dascyllus abudafur* associated with the placebo-treated coral colonies of *Pocillopora verrucosa*.
